# Mr. William Ash

**Published:** 1904-01

**Authors:** 


### Obituary.

### MR. WILLIAM ASH.

Born April 18, 1819; died November 19, 1903.

We regret to announce that Mr. William Ash, the youngest son of Claudius Ash, the founder of the firm of Claudius Ash & Sons, died at his London residence, Tower House, Camden Road, N. W., on Thursday, November 19, in his eighty-fifth year. Born in the parish of St. James's, Westminster, on the 18th of April, 1819, he was educated in London, and in due course joined his father and his brothers George, Claudius, and Edward in business.

For nearly sixty years Mr. William Ash was a familiar figure at Broad Street, and all who remember him will recall his kindly and urbane manner. He ceased to take an active part in the business about ten years ago.

In addition to the business side of his life, Mr. William Ash was deeply interested in the dental profession, and was very closely associated with the Dental Hospital of London from its inception. For a great many years he was an active member of the committee of that institution, and took a prominent part in its removal from Soho Square to Leicester Square, the result of a proposal by Mr. (afterwards Sir) Edwin Saunders. At the annual general meeting of the governors of the hospital in 1873, Mr. Ash proposed an alternative to the Saunders plan. "His idea was to build suitable premises, rather than to convert old ones into the nearest approach to convenience they would allow. At considerable trouble he had procured plans and drawings in connection with sites to be obtained from the Metropolitan Board of Works, on which a hospital could be erected, adapted in all points to the requirements of the managing and medical staff, the students, and also the Odontological Society. . . . His proposal was discussed, but eventually the decision was given in favor of the original plan."[1]

Soon after his twenty-first birthday Mr. William Ash married Sarah, the daughter of Mr. James Matchwick, of Guildford, Surrey, who predeceased him by only sixteen days.

[1] Hill's History of the Reform Movement in the Dental Profession in Great Britain, p. 237.

Three years ago a stained glass window was inserted in the church at Heathfield, Sussex, in which parish Mr. Ash had a country residence, as a thank-offering, and in commemoration of his diamond jubilee.

Mr. William Ash leaves three sons and three daughters. Of the sons, William Henry and Claudius James are at present the senior Directors of the firm of Claudius Ash & Sons, Limited.

The funeral took place on Monday, November 23, at Highgate Cemetery and was largely attended by members, relatives, and friends of the family; also by many of the employees at Broad Street and some from the Factory, Kentish Town.

